# Intrapetrous Internal Carotid Artery Pseudoaneurysm Secondary to Malignant Otitis Externa: A Case Report

**DOI:** 10.7759/cureus.110046

**Published:** 2026-06-01

**Authors:** Younes Abdourabbih, Mokhtar EL Mekhtoume, Hajar Ouazzani, Ismail Chaouche, Amal Akammar, Meriem Haloua, Badreddine Alami, Moulay Youssef Lamrani, Meryem Boubbou, Mustapha Maaroufi, Nizar El Bouardi

**Affiliations:** 1 Radiology, Hassan II University Hospital Center of Fez, Sidi Mohamed Ben Abdallah University, Fez, MAR; 2 Mother and Child Radiology, Hassan II University Hospital Center of Fez, Sidi Mohamed Ben Abdallah University, Fez, MAR

**Keywords:** external malignant otitis, infection, intra-petrous internal carotid artery, pseudoaneurysm, skull base

## Abstract

Malignant otitis externa (MOE) can rarely progress to skull base osteomyelitis (SBO) with catastrophic vascular complications. We describe a 68-year-old man with uncontrolled diabetes (HbA1c 9%) who presented with left otalgia, purulent otorrhea, and hearing loss. Initial workup showed CRP 23 mg/L, leukocytes 8,000/µL, and ear pus culture positive for Pseudomonas aeruginosa. Computed tomography (CT) and magnetic resonance imaging (MRI) demonstrated MOE with temporal bone osteomyelitis. Despite prolonged intravenous antibiotics (imipenem, colistin) and topical ofloxacin, symptoms persisted. Three months later, he developed a headache, pulsatile tinnitus, and a pulsatile retro‑tympanic mass; MRI and 3D time-of-flight (TOF) angiography revealed progression of SBO with a pseudoaneurysm arising from the intrapetrous segment of the left internal carotid artery and ipsilateral sigmoid sinus septic thrombosis. Digital subtraction angiography was planned for definitive characterization and endovascular management, but the patient deteriorated rapidly and died of overwhelming septic shock before intervention. This case highlights that MOE can extend to involve the petrous internal carotid artery (ICA) and produce infected pseudoaneurysms; early vascular imaging [computed tomography angiography (CTA)/ magnetic resonance angiography (MRA) and digital subtraction angiography (DSA) when indicated], aggressive antimicrobial therapy, and prompt multidisciplinary planning for endovascular or surgical intervention are essential to reduce morbidity and mortality.

## Introduction

Malignant otitis externa (MOE) is an aggressive infection of the external auditory canal and adjacent skull base that primarily affects elderly and immunocompromised patients, most notably those with poorly controlled diabetes mellitus. Pseudomonas aeruginosa is the most commonly isolated pathogen, although polymicrobial and fungal infections have been reported [1,2]. The infection typically begins in the external auditory canal and spreads to the temporal bone via Santorini fissures and Haversian canals, producing skull base osteomyelitis (SBO) that may involve the petrous apex, clivus, and adjacent neurovascular structures [1-3]. Complications of SBO include cranial neuropathies (CN), particularly CNs VII, VI, and V, venous sinus thrombosis, cerebral infarction, and vascular wall involvement leading to mycotic or infected pseudoaneurysms of the internal carotid artery (ICA) [3]. Infected ICA pseudoaneurysms result from the contiguous spread of infection with enzymatic degradation and ischemic injury of the arterial wall, and they carry a high risk of rupture and hemorrhage; early recognition and combined medical-endovascular or surgical management are therefore critical [4].

## Case presentation

A 68-year-old man with poorly controlled diabetes (HbA1c 9%) presented with left ear pain, purulent otorrhea, and hearing loss. Initial workup showed C-reactive protein (CRP) 23 mg/L (normal 0-5 mg/L), leukocytes 8,000/µL (normal 4,000-10,000/µL), and pus culture positive for Pseudomonas aeruginosa. Computed tomography (CT) revealed malignant otitis externa with skull base osteomyelitis (Figure 1). Otoscopy demonstrated a narrowed left external auditory canal with an inflammatory polyp and a raspberry‑like pulsatile mass in the tympanic cavity; otoscopic images are not available. Despite intravenous antibiotics (imipenem, colistin) and topical ofloxacin, his condition failed to improve. Three months later, he developed a persistent headache and pulsatile tinnitus. Magnetic resonance imaging (MRI) demonstrated progression of skull base osteomyelitis with phlegmonous infiltration and collections at the skull base (Figure 2). Vascular sequences [3D time-of-flight (TOF) and post-contrast MRI] identified a focal high‑flow outpouching from the intrapetrous segment of the left internal carotid artery consistent with a pseudoaneurysm, and absence of flow signal/opacification of the left sigmoid sinus compatible with septic thrombosis (Figures 3, 4). Digital subtraction angiography was planned for definitive characterization and endovascular management, but the patient rapidly deteriorated and died of severe septic shock before intervention.

**Figure 1:**
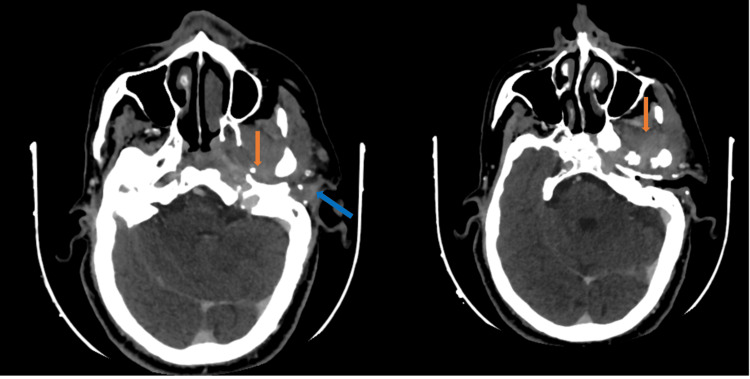
Head CT in axial plan with contrast injection (A and B) Hypodense filling of the external ear with thickening and enhancement of its walls (blue arrow), along with filling of the middle ear and a hypodense infiltration at the skull base (orange arrow). CT: computed tomography.

**Figure 2:**
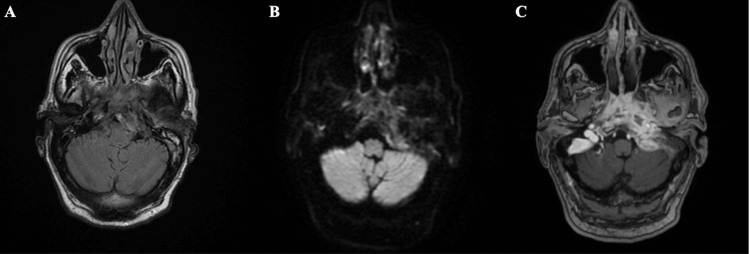
MRI in axial view: FLAIR sequence(A), DWI (B), T1 post contrast injection sequence (C) Phlegmonous infiltration in the retropharyngeal space extending to the skull base, containing collections within it, with high signal intensity in the FLAIR sequence and a mild restriction of diffusion. MRI: magnetic resonance imaging, FLAIR: fluid-attenuated inversion recovery.

**Figure 3:**
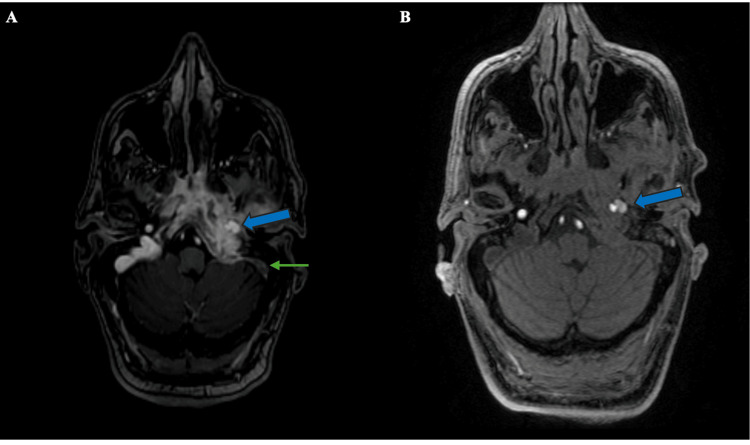
MRI in axial projection (A): T1 post post contrast injection sequence, (B): 3D TOF Pseudoaneurysm originating from the intrapetrous portion of the left carotid artery (blue arrow), Absence of opacification of the left sigmoid sinus, consistent with septic thrombosis (green arrow). MRI: magnetic resonance imaging, TOF: time-of-flight.

**Figure 4:**
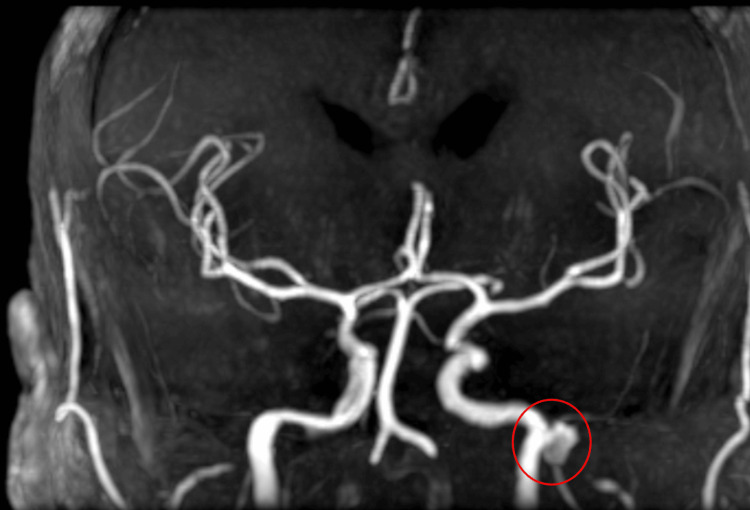
3D TOF in coronal projection Pseudoaneurysm originating from the intrapetrous portion of the left carotid artery (red circle). TOF: time-of-flight.

## Discussion

MOE is an aggressive skull base infection that occurs mainly in immunocompromised patients, particularly those with uncontrolled diabetes. Pseudomonas aeruginosa is the predominant pathogen, although other bacteria and fungi have also been reported [1,2]. The infection spreads from the external auditory canal through the fissures of Santorini and Haversian canals into the temporal bone, producing SBO. Focal involvement of the petrous apex can lead to petrous apicitis, which may present as Gradenigo syndrome - the classic triad of retro-orbital pain from trigeminal involvement, abducens nerve palsy, and persistent otorrhea. These localized processes increase the risk of contiguous neurovascular complications, including cranial neuropathies, venous sinus thrombosis, cerebral infarction, and arterial wall involvement with pseudoaneurysm formation [3,4].

Infected pseudoaneurysms arise from direct extension of osteomyelitis or abscess into the arterial wall with destruction of the adventitia and media; mechanisms include proteolytic degradation by bacterial enzymes and inflammatory cells, vasa vasorum thrombosis with ischemic weakening, and focal necrosis from mass effect or abscess, producing a contained defect communicating with the arterial lumen (pseudoaneurysm) with high rupture risk [4-6]. Clinically, infected ICA pseudoaneurysms may present with mass effect, cranial neuropathies, or hemorrhagic manifestations such as epistaxis or otorrhagia; otorrhagia can be the first sign of a middle ear or petrous ICA aneurysm [7]. CT angiography or MR angiography are useful, but digital subtraction angiography (DSA) remains the diagnostic gold standard and guides endovascular planning; rapid systemic deterioration from sepsis (as in this case) can preclude definitive vascular treatment and increase mortality [8].

Treatment of infected petrous ICA pseudoaneurysms requires prompt, individualized, multidisciplinary care combining aggressive antimicrobial therapy and tailored vascular intervention. Endovascular approaches are often preferred given the deep skull base location. Coil embolization can achieve immediate sac thrombosis and may preserve the parent artery in select anatomies, but risks migration, recanalization, thromboembolism, and intraprocedural rupture, especially with broad-neck or heavily infected lesions [6]. Balloon test occlusion followed by permanent parent artery occlusion definitively eliminates flow when collateral circulation is adequate but requires demonstration of ischemic tolerance and carries stroke risk if collaterals fail [4]. Covered stents immediately exclude the pseudoaneurysm while preserving flow but require dual antiplatelet therapy and risk device infection, endoleak, or migration, and are limited by tortuous petrous anatomy [9]. Flow-diverting stents offer a reconstructive option for wide-neck or fusiform lesions by promoting gradual thrombosis and vessel remodeling but require dual antiplatelet regimens, produce delayed occlusion, and carry risks of in-stent thrombosis or device infection, complicating use during active sepsis [10]. Because implanted devices in infected environments risk persistent device infection and recurrence, endovascular intervention should be accompanied by targeted, often prolonged, intravenous antibiotics and, when feasible, surgical debridement or abscess drainage to reduce infectious burden and improve outcomes [4,6]. Case reports and series document successful endovascular exclusion of petrous ICA pseudoaneurysms arising from otogenic or skull base infections using coils, parent vessel occlusion, covered stents, and flow diverters, but also report complications including recurrent infection, rebleeding, ischemic stroke, and device-related issues, underscoring the need for individualized, multidisciplinary management and close follow-up [7-10].

## Conclusions

MOE can rarely cause infected intrapetrous ICA pseudoaneurysms with catastrophic outcomes. Clinicians should maintain a high index of suspicion for vascular complications in diabetics with MOE who develop pulsatile tinnitus, retro‑tympanic masses, or new neurologic signs. Prompt vascular imaging [computed tomography angiography (CTA)/ magnetic resonance angiography (MRA) followed by DSA as indicated) and early multidisciplinary planning for combined aggressive antimicrobial therapy and timely endovascular or surgical intervention is essential to reduce morbidity and mortality.
